# Modified Lautenbach technique in the treatment of an open infected non-union of the clavicle—a case report

**DOI:** 10.3109/17453674.2012.665332

**Published:** 2012-06-04

**Authors:** Benjamin Johnson, Peter Thomas, Damian McClelland

**Affiliations:** The Trauma and Orthopaedic Department, University Hospital of North Staffordshire, Stoke on Trent, UK; Correspondence: bjohnso25@yahoo.co.uk

In 2009, a 26-year-old man presented at his local hospital with a closed displaced mid-shaft clavicle fracture following a fall from his mountain bike. There was no neurovascular compromise. He underwent open reduction and internal fixation of the clavicle 1 week later, with an anterior reconstruction plate. The wound healed with no signs of infection.

14 months after fixation, ulceration developed over the plate, which was removed and the wound was debrided and closed. 1 week later, he tripped from a standing height and landed on the same shoulder. Radiographs revealed non-union and osteopenia, and the ends of bone were protruding through the skin (Figure 1 and 2). The white cell count was 13, the CRP was 57, and the ESR was 15. The wound was debrided and treatment with intravenous benzylpenicillin (1.2 g) and flucloxacillin (2 g) was begun. He was referred on to our unit and was again operated on. The bone ends were again debrided and dead tissue excised. The bone ends were opposed and a Pennig external fixator (Orthofix, Srl, Italy) applied with 2 medial and 2 lateral pins (Figure 3). The Lautenbach technique, the use of a closed double-lumen suction irrigation system, generally involves intramedullary placement of the irrigation tubes. However, as it is not possible to ream the clavicle to a sufficient diameter to allow this, we used a modification of the Lautenbach technique and left the end of the irrigation tube at the fracture site (Figure 3).

**Figure 1.**
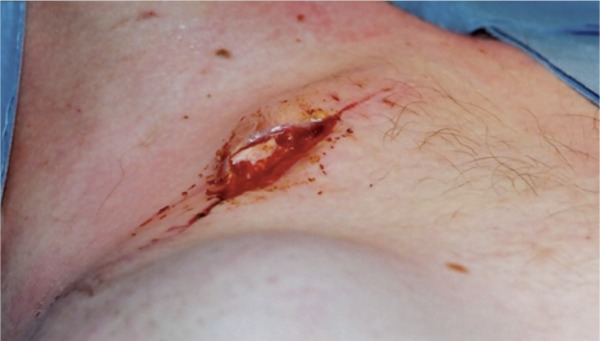
Protrusion of the medial aspect of the clavicle through the skin.

**Figure 2.**
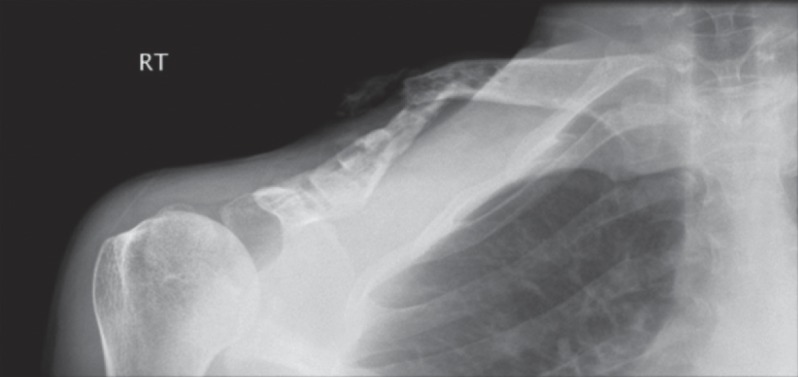
Radiograph showing patchy osteopenia, erosions, and no evidence of union.

**Figure 3.**
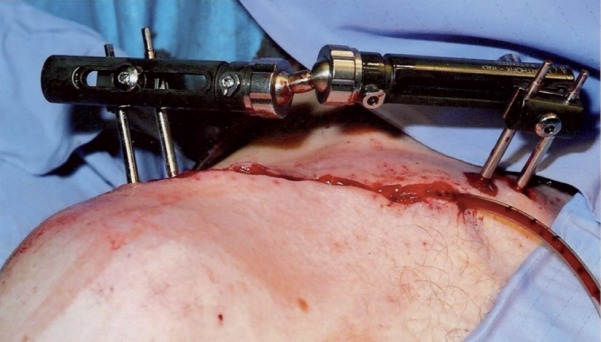
Pennig external fixator and Lautenbach drain emerging from the closed wound.

The closed double-lumen suction irrigation system was constructed by threading an epidural catheter through the connector of an intravenous giving set (the connecting pipe to a bag of fluid). A low-pressure drain was then connected. This created a double lumen with a fine internal catheter for the delivery of antibiotics and thrombolytic agents and the wider surrounding catheter for intermittent suction.

Every 4 hours, the drain was clamped—allowing administration of teicoplanin (8 mg), gentamicin (10 mg), and streptokinase (2,000 units). 1 mL of normal saline was injected prior to each injection and following the last injection, to maintain patency of the tubes. The drain was then left clamped for 20 min before recommencing free drainage. Intravenous flucloxacillin (2 g) and benzylpenicillin (1.2 g) were also given.

Coagulase-negative *Staphylococcus aureus* that was sensitive to flucloxacillin was isolated on culture from the deep tissue samples taken in theater. After 1 week, the Lautenbach drain was removed. The patient was discharged on oral flucloxacillin (1 g, 4 times daily) and penicillin V (500 mg, 4 times daily) for 6 weeks postoperatively.

The external fixator was removed 3.5 months after it was applied. Clinically and radiographically, the fracture had united (Figure 4). At 6 months postoperatively, he had regained full motion in his shoulder and radiographs showed union of the fracture (Figure 5)

**Figure 4.**
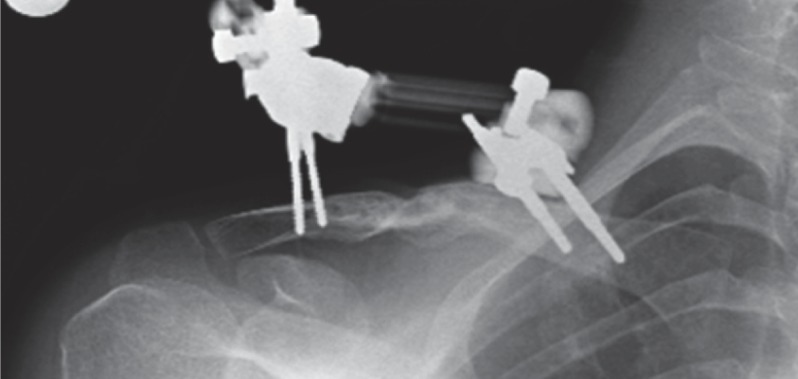
Radiograph of the clavicle 3.5 months after application of the external fixator, showing callus formation at the fracture site.

**Figure 5.**
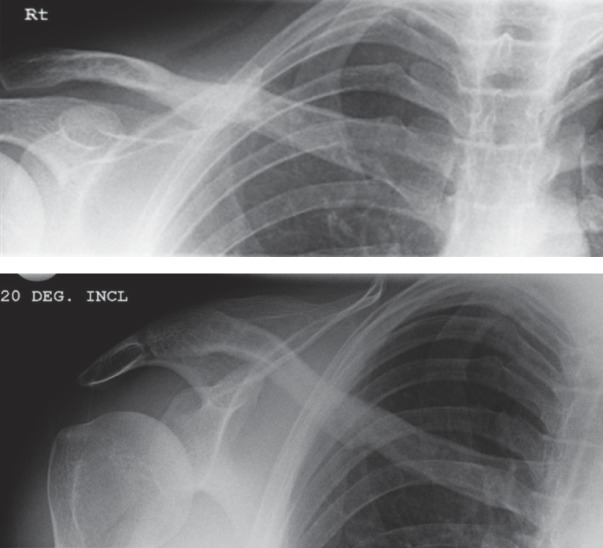
AP (top) and 20-degree (bottom) radiographs of the clavicle 6 months after surgery, showing union of the clavicle fracture.

## Discussion

Deep infection after internal fixation of clavicle fractures is associated with a poor rate of union and poor functional outcome (Duncan et al. 2005). Shen et al. (1999) described 1 deep infection in 232 plated clavicle fractures. Böstman et al. (1997) reported 8 infections in 103 cases treated with plate fixation. 5 of these were deep infections.

Deep infections following open reduction and internal fixation of clavicle fractures will become more common, as a greater proportion of clavicle fractures are being treated surgically. There is little in the literature regarding the management of deep infections of the clavicle. Duncan et al. (2005) recommended thorough debridement of all necrotic tissue and removal of all non-absorbable sutures and metal work followed by at least 4–6 weeks of intravenous antibiotics and an additional period of treatment with oral antibiotics. However, only 2 of the 6 patients reported had achieved union, 1 of which required a periosteal vascularised bone graft.

External fixators have been used in the treatment of clavicle fractures for many years (Schuind et al. 1988, Strauss et al. 2008, Bonnevialle et al. 2010), with the main indications being open fracture or infected non-union.

In 1986, Lautenbach described a continuous local antibiotic delivery system for the treatment of infected total hip replacements (Weber and Lautenbach 1986). Its use has also been described in the treatment of posttraumatic osteomyelitis (Hashmi et al. 2004, Caesar et al. 2009). The Lautenbach system involves the use of a closed double-lumen tube delivering antibiotics locally, followed by suction. The suction element of the system allows the microbiology, volume, and appearance of the drainage fluid to be checked. It also allows obliteration of the dead space created by soft tissue and bone debridement.

There have been no previous publications in which the Lautenbach technique has been used in the management of infected non-unions of the clavicle. We used a modified Lautenbach technique, as the clavicle cannot be reamed in the same way as the tibia and femur to accommodate the irrigation tubes, which are traditionally inserted in an intramedullary fashion. Instead, the end of the double-lumen suction irrigation system was placed at the fracture site, allowing local delivery of antibiotics and antithrombotic agents. Hashmi et al. (2004) and Caesar et al. (2009) used the suction irrigation system for approximately 3 weeks. We removed the double-lumen tube on day 7, as the inflammatory markers had markedly improved and the patient was suitable for discharge with appropriate oral antibiotic treatment.

Deep infection of the clavicle will become increasingly common, as clavicle fractures are more often being treated by open reduction and internal fixation. We believe that the use of a modified Lautenbach system in conjunction with external fixation may be of value in the management of infected clavicle non-unions.
